# Abnormal Functional and Structural Connectivity of Amygdala-Prefrontal Circuit in First-Episode Adolescent Depression: A Combined fMRI and DTI Study

**DOI:** 10.3389/fpsyt.2019.00983

**Published:** 2020-02-05

**Authors:** Feng Wu, Zhaoyuan Tu, Jiaze Sun, Haiyang Geng, Yifang Zhou, Xiaowei Jiang, Huizi Li, Lingtao Kong

**Affiliations:** ^1^ Department of Psychiatry, The First Affiliated Hospital of China Medical University, Shenyang, China; ^2^ Department of Radiology, The First Affiliated Hospital of China Medical University, Shenyang, China; ^3^ Department of Gerontology, The First Affiliated Hospital of China Medical University, Shenyang, China; ^4^ Brain Function Research Section, The First Affiliated Hospital of China Medical University, Shenyang, China

**Keywords:** adolescent major depressive disorder, functional connectivity, magnetic resonance imaging, diffusion tensor imaging, amygdala, ventral prefrontal cortex

## Abstract

**Background:**

Abnormalities of functional and structural connectivity in the amygdala-prefrontal circuit which involved with emotion processing have been implicated in adults with major depressive disorder (MDD). Adolescent MDD may have severer dysfunction of emotion processing than adult MDD. In this study, we used resting-state functional magnetic resonance imaging (rs-fMRI) and diffusion tensor imaging (DTI) to examine the potential functional and structural connectivity abnormalities within amygdala-prefrontal circuit in first-episode medication-naïve adolescents with MDD.

**Methods:**

Rs-fMRI and DTI data were acquired from 36 first-episode medication-naïve MDD adolescents and 37 healthy controls (HC). Functional connectivity between amygdala and the prefrontal cortex (PFC) and fractional anisotropy (FA) values of the uncinate fasciculus (UF) which connecting amygdala and PFC were compared between the MDD and HC groups. The correlation between the FA value of UF and the strength of the functional connectivity in the PFC showing significant differences between the two groups was identified.

**Results:**

Compared with the HC group, decreased functional connectivity between left amygdala and left ventral PFC was detected in the adolescent MDD group. FA values were significant lower in the left UF within the adolescent MDD group compared to the HC group. There was no significant correlation between the UF and FA, and the strength of functional connectivity within the adolescent MDD group.

**Conclusions:**

First-episode medication-naïve adolescent MDD showed decreased functional and structural connectivity in the amygdala-prefrontal circuit. These findings suggest that both functional and structural abnormalities of the amygdala-prefrontal circuit may present in the early onset of adolescent MDD and play an important role in the neuropathophysiology of adolescent MDD.

## Introduction

Major depressive disorder (MDD) is characterized by emotional dysregulation, implicating abnormalities of frontal-limbic neural circuits involved in emotional processing as the core feature. Convergent studies provide consistent evidence for functional and structural abnormalities in the prefrontal cortex (PFC) and amygdala, the key components of frontal-limbic neural circuits in adult MDD. Adolescent MDD is associated with the prominence of irritability, mood reactivity, and fluctuating symptoms (1), reflecting possible severer emotional processing dysfunction than adult MDD. Furthermore, as its strong links with recurrence later in life (2), investigation of depression in adolescence may help us to further understand the role of abnormal developmental process leading to adult MDD.

Dysfunction of amygdala-prefrontal circuits has been implicated in MDD through functional magnetic resonance imaging (fMRI). Hyperactivation of amygdala and hypoactivation of PFC were shown in task-related fMRI studies within adult MDD, as well as adolescent MDD. Recently, resting-state fMRI (rs-fMRI) was used to investigate resting state functional connectivity (rsFC), the correlation of low frequency blood oxygen level-dependent signal fluctuations between brain regions in MDD (3). Our previous study demonstrated decreased amygdala-PFC functional connectivity in adult MDD (4). Other researchers also reported similar findings (5, 6), suggesting that dysfunction of these circuits may play an important role in the neuropathophysiology of MDD. Correspondingly, few studies detected functional connectivity between amygdala and other brain regions in adolescent MDD. Connolly et al’s study reported decreased amygdala-dorsolateral prefrontal cortex (DLPFC) functional connectivity and amygdala-ventromedial prefrontal cortex (VMPFC) functional connectivity in adolescent MDD patients (7). Cullen et al’s two studies failed to find amygdala-PFC functional connectivity abnormalities, but find decreased functional connectivity in amygdala-limbic networks and anterior cingulate cortex (ACC)-based networks (8, 9). Luking et al.’s another study reported reduced amygdala functional connectivity with dorsal frontal/parietal and limbic regions in children with MDD history (10). Taken together, inconsistent findings suggest that functional connectivity of amygdala-related circuits need to be further investigated in adolescent MDD.

As white matter fibers structural connecting brain regions into neural circuits, disconnection of white matter fibers may provide the structural basis of functional connectivity abnormalities in the brain (11). Diffusion tensor imaging (DTI) is a MRI technique for detecting white matter microstructure integrity *in vivo*. Fractional anisotropy (FA) which measures the principal directionality of water diffusion is the commonly used parameter to assess whiter matter integrity in DTI studies. Decreased FA values were detected in the uncinate fasciculus, the superior longitudinal fasciculus, the cingulum, the corpus callosum (12), the genu and UF (13) in adult MDD, indicating abnormalities of white matter fiber integrity. In the recent studies of adolescent MDD, Cullen et al. reported lower FA in the tract connecting subgenual ACC to amygdala (14), Bessette et al. reported lower FA in corpus callosum, midbrain white matter tracts, and corticospinal tracts (15), while Aghajani et al. reported lower FA in the body of the corpus callosum, as well as higher FA in the uncinate fasciculus (16), Taken together, these findings support the hypothesis that white matter deficits of frontal-limbic neural circuits may present in the early stage of depression.

The relationship between structural and functional connectivity has been noticed. Kim et al. reported a positive relationship between amygdala reactivity and white matter integrity of the uncinate fasciculus in healthy controls, suggesting the relationship between functional and structural connectivity in amygdala-prefrontal circuits (17). Furthermore, negative relationship between amygdala volume and activity during emotional processing tasks in MDD (18). The positive correlations between FA values of left UF and resting state functional connectivity of the left vlPFC-amygdala and the left vlPFC-hippocampus in late life depression (19). One previous study reported that amygdala volume changes is negative to the age of on set, and younger MDD adults (< 30 years old) show more abnormal FC changes in left amygdala, while older MDD adults (≥30 years old) are in right amygdala (20). However, the relationship between functional and structural connectivity in adolescent MDD is not fully explored. Hence, we combined rs-fMRI and DTI in the current study to examine the functional and structural connectivity and their relationship within amygdala-prefrontal circuits in first-episode medication-naïve MDD adolescents. In our hypothesis, functional and structural connectivity abnormalities would be detected between amygdala and PFC within adolescent MDD, as well as an association between the functional and structural connectivity in this circuitry.

## Materials and Methods

### Participants

We recruited 36 medication-naïve adolescent outpatients with MDD from the outpatient clinic of the Department of Psychiatry and 37 healthy control subjects (HC) matched for sex, age and education by advertisements in the Frist Affiliated Hospital of China Medical University. A trained psychiatrist individually diagnosed all participants using the Schedule for Affective Disorders and Schizophrenia for School-Age Children (KSADS-PL). All MDD patients met the following inclusion criteria: fulfilling KSADS-PL criteria for MDD; first depressive episode; onset age between 13 and 17; no comorbid diagnosis of other affective and psychotic disorders; We used the 17-item Hamilton Depression Rating Scale (HAMD-17) (21) to score the severity of depression. All HC adolescents were confirmed the absence of psychiatric disorders. Any HC was excluded if he/she had any family history of psychiatric disorders in their first- or second-degree relatives. Any participant with the following additional criteria was excluded: history of head injury or neurological disorder; history of drug abuse or dependence; contraindications for MRI. All participants were scanned within 24 h of initial contact, and rated on the HAMD-17 at the time of scanning.

The study was approved by the Institutional Review Board of the China Medical University. All participants and their parent/legal guardian received a detailed description of the study, after that, they provided written informed consent to make sure they fully understand and agree their involvement of the study.

### Mri Data Acquisition

A GE Signa HDX 3.0T MRI scanner (General Electric, Milwaukee, USA) with a standard head coil at the First Affiliated Hospital of China Medical University was used to perform the magnetic resonance imaging scan. Restraining foam pads and ear plugs were used for each participant to minimize the head motion and reduce the noise interference during the scan. Participants were asked to remain awake throughout the scan and keep their eyes closed. We used a gradient-echo planar imaging sequence to collect the rs-fMRI data with the following scan parameters: TR 2,000 ms, TE 30ms, 35 contiguous axial slices, 3 mm thickness, without gap, matrix 64 × 64, FOV 240 × 240mm^2^, flip angle 90°. We used a spin-echo planar imaging sequence to collect the DTI data with the following scan parameters: 25 non-collinear directions, b = 1,000 s/mm^2^, TR 17,000 ms, TE 85.4 ms, 65 contiguous axial slices, 2 mm thickness, without gap, imaging matrix 120 × 120, FOV = 240 × 240 mm^2^.

### Rs-fMRI Data Processing

We used Resting-State fMRI Data Analysis Toolkit (REST) with Statistical Parametric Mapping 8 (SPM8) to processed the rs-fMRI data. The first 10 volumes of scanned data of each participant were deleted due to magnetic saturation effects. The remaining images were preprocessed with the following steps: First, slice timing and head motion correction: head motion parameters were computed by estimating translation in each direction and the angular rotation about each axis for each volume. The rs-fMRI data was excluded if their head motion was >2 mm maximum displacement in any of the x, y, or z directions or 2° of any angular motion throughout the course of the scan (no participants were excluded). Second, spatial normalization image to the standard Montreal Neurological Institute (MNI) space and resampled voxel size into 3 × 3 × 3 mm^3^ voxels then smoothing with a Gaussian filter of 6 mm full-width at half-maximum (FWHM). Then REST software was used to remove linear drift through linear regression and temporal band-pass filtering (0.01–0.08 Hz) to reduce the effects of low-frequency drifts and physiological high-frequency noise. Linear regression of head motion parameters, global mean signal, white matter signal and cerebrospinal fluid signal was performed to remove the effects of the nuisance covariates.

The left and right amygdala ROIs were defined separately according to the automated anatomical labeling (AAL) template contained in REST (22). The time course for all voxels of each ROI were averaged to calculate the mean time course for each amygdala ROI. The time course of each amygdala ROI was then correlated with the time course of each pixel in the brain, resulted with a correlation map for each subject that contained the correlation coefficient for each voxel with that of the amygdala ROI. The resulting correlation coefficients were transformed into z-scores. Subject-specific maps of resting state correlations to each amygdala ROI were created.

### DTI Data Processing

We processed DTI data using the PANDA toolbox (23) in FSL diffusion toolkit and MRIcron. DICOM files were first converted into NIfTI images, then estimate the brain mask, crop images, correct for the eddy-current effect, average acquisitions, and calculate DTI metrics. Finally, diffusion metrics were produced ready for statistical analysis. The individual diffusion metric images were transformed from native space into a standard Montreal Neurological Institute (MNI) space (voxel size 1mm×1mm×1mm^3^) *via* spatial normalization. The ICBM-DTI-81 WM labels atlas in the standard space allow for parcellation of the entire white matter into multiple regions of interest (ROI) (24). PANDA toolbox was used to calculate the regional diffusion metrics by averaging the values within each region of the WM atlases. These resultant ROI-based data was statistically analyzed with SPSS and other statistical packages. In our study, we selected left and right uncinate fasciculus (UF) as the ROIs.

### Statistical Analysis

We used the independent two-sample t tests and χ^2^ tests compare demographic data and HAMD scores between the MDD and HC groups with SPSS 22.0. Two-tailed values of P < 0.05 were considered statistically significant.

The subject-specific maps of resting state correlations from the amygdala to all brain voxels of rs-fMRI data were combined across subjects within the MDD group and within the HC group. With age as covariate, Voxel-based 1-sample t-tests were used to produce group whole-brain composite maps. Then we created the contrast maps to assess between-group differences using voxel-based 2-sample (MDD vs. HC) t-tests. The contrast maps were corrected for multiple comparisons using Monte Carlo simulation within the PFC which was our hypothesized region. We defined the PFC ROI including 20 labels of AAL template, corresponding to Brodmann areas (BA) 9, 10, 11, 12, 24, 25, 32, 44, 45, 46, 47. The contrast map threshold was set at p < 0.05 for each voxel, with cluster size of at least 32 voxels (864mm^3^), corresponding to the p < 0.05 corrected by AlphaSim.

We used two sample t-tests to compare group differences in the FA values separately for the atlas-based ROIs in SPSS. Two-tailed values of p < 0.05 were considered statistically significant.

To further evaluate the relationship between functional and structural connectivity, we investigated the correlations in MDD and HC participants separately between the FA values of UF and the strength of the functional connectivity in the regions showing significant differences between the two groups. Pearson’s correlation analysis was used, and statistical significance was set at 0.05 (two-tailed).

## Results

### Demographic and Clinical Scales

There were no significant differences in age (p = 0.972), gender (p = 0.236),or education (p = 0.228) between the adolescent MDD and the HC groups. MDD adolescents had significantly higher HAMD scores than the HC (p < 0.001, **Table 1**).

**Table 1:** Demographic and clinical data of participants.

Characteristic	MDD	HC	Statistic	p-value
Number	36	37		
Age (year, mean ± SD)	15.6 ± 1.27	15.6 ± 1.30	t = 0.03	0.972
Gender (male/female)	12/24	18/19	χ^2^^=^ 1.768	0.236
Education (year, mean ± SD)	9.86 ± 1.46	10.03 ± 1.63	t = 1.217	0.228
HAMD-17 score(mean ± SD)	23.19 ± 7.54	1.41 ± 1.64	t = 17.161	0.000*
Illness duration (month, mean ± SD)	10.11 ± 10.61	NA	NA	NA

*p < 0.05 SD, standard deviation.

MDD, major depression disorder.

HC, healthy controls.

HAMD, Hamilton Depression Rating Scale.

NA, not applicable.

### Between-Group Differences in rsFC and DTI

**Figure 1:**
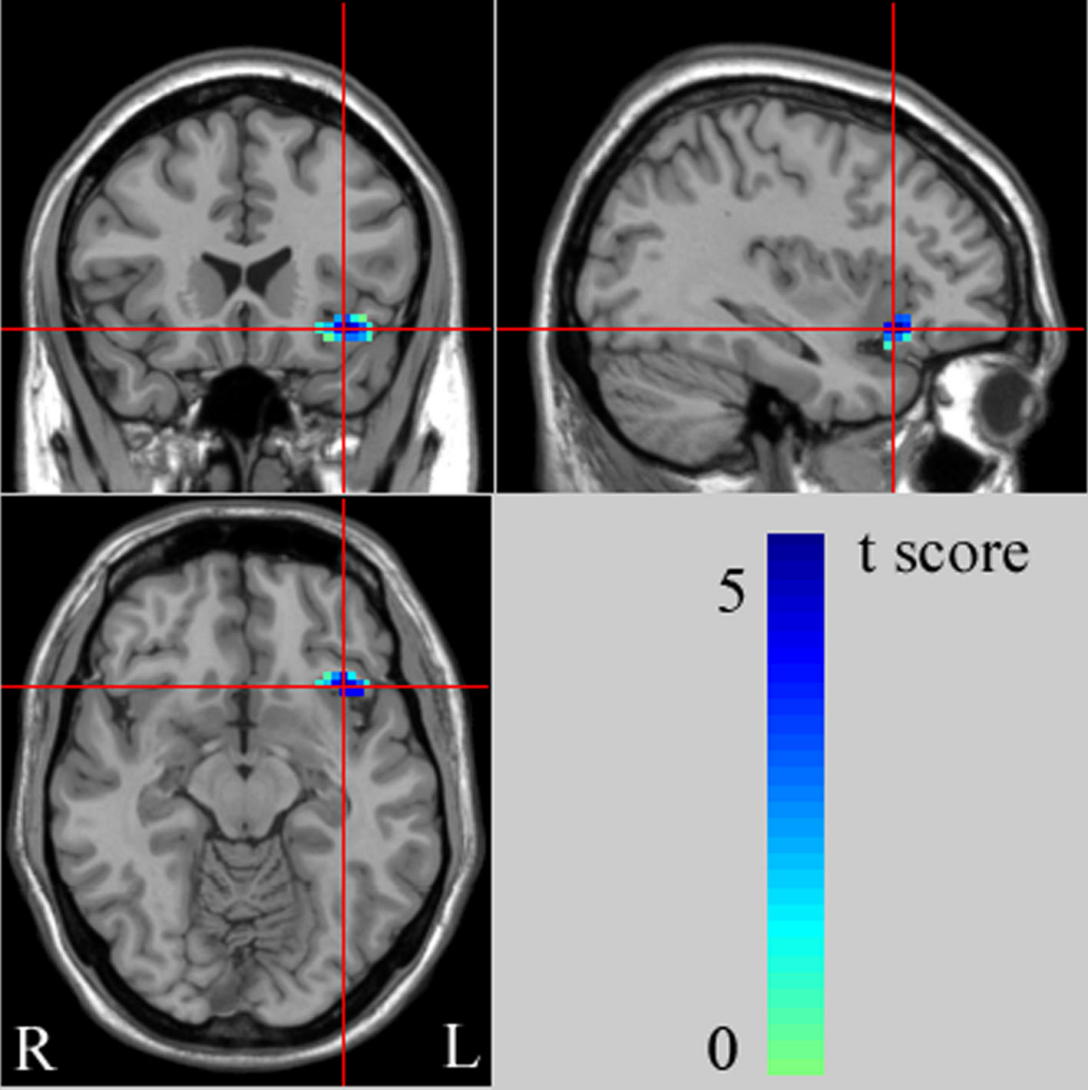
The images (MNI coordinate x = -35mm, y = 21mm, z = -11mm) display the regions in left ventral prefrontal cortex (VPFC) that show decreased functional connectivity from the left amygdala in adolescents with major depressive disorder (MDD), compared to healthy controls (HC) at rest. The color bar represents the range of T values. L, left brain; R, right brain.

With age as covariant We found significantly decreased left ventral PFC (VPFC, BA 47) rsFC from the left amygdala in the adolescent MDD group, compared with the HC group [peak MNI coordinates of the left VPFC region: x = -35, y = 21, z = -11, 45 voxels (1215mm^3^), T = 3.53] (**Figure 1**, **Table 2**). This finding correspond to a corrected P < 0.05 by AlphaSim correction. Compared with the HC group, the MDD group showed no significant region in rsFC between the right amygdala and PFC regions.

**Table 2:** Regions with decreased rsFC from the amygdala in subjects with major depressive disorder compared to health control subjects.

Regions	BA	Peak MNI coordinate			Voxel size	t value	P value
		X	Y	Z		
Left VPFC	47	-35	21	-11	45	3.53	<0.05

rsFC, Resting state functional connectivity.

VPFC, ventral prefrontal cortex.

BA, Brodmann areas.

MNI, Montreal Neurological Institute.

Compared with healthy controls, MDD group had significant decreased FA values in the left UF. There were no significant findings of right UF in MDD relative to controls (**Table 3**).

**Table 3:** Two sample t test results for FA values of left and right UF.

Regions	FA (mean ± SD)			t value	P value
	MDD (n = 36)	HC(n = 37)		
Left UF	0.382 ± 0.02	0.392 ± 0.02	2.059	0.043*
Right UF	0.388 ± 0.03	0.392 ± 0.02	0.678	0.500

*t < 0.05UF, uncinate fasciculus.

SD, standard deviation.

MDD, major depression disorder.

HC, healthy controls.

### Correlation Between FA and the Strength of Functional Connectivity

In post-hoc correlation analyses, no significant association was detected between FA value of the left UF and the strength of rsFC of VPFC from the left amygdala in the MDD group (r = -0.377, P = 0.058). There was also no significant correlation in the HC group (r = -0.118, P = 0.574).

## Discussion

In this study, we reported decreased rsFC between left amygdala and left VPFC in adolescent MDD compared to HC. Furthermore, deficits of white matter integrity in the left uncinate fasciculus, fiber tracts connecting VPFC to temporal regions (including amygdala and hippocampus) were detected in adolescent MDD compared to HC. To our knowledge, this is the first study using differential MRI methods to explore functional and structural connectivity abnormalities in the amygdala-prefrontal circuits within adolescent MDD. Our findings provide primary evidence implicating abnormalities of amygdala-prefrontal circuits as the key components in the pathophysiology of adolescent MDD.

Our results of decreased rsFC between left amygdala and left VPFC in adolescent MDD (age 13–17) compared to HC are consistent with our previous findings of both rs-fMRI and task-fMRI in adult MDD (age 18–45) (4, 25). Amygdala-VPFC circuits play an important role in emotion processing (26). Dysfunction of amygdala and ventral frontal regions including VPFC and subgenual ACC were widely reported in adult MDD. Recently, several fMRI studies with adolescent MDD also focused on the function of amygdala and related circuits mediating emotion processing. Hyperactivation of amygdala and ACC were shown during facial-emotion matching task within adolescent MDD (27). In rs-fMRI studies, Cullen et al. reported decreased functional connectivity in the subgenual ACC-based network (8) and amygdala-hippocampus/brainstem circuits (9), but failed to find amygdala-frontal functional connectivity abnormalities. Taken together, our findings suggest that deficits of amygdala-prefrontal functional connectivity may emerge in the early onset of MDD and reflect the emotional dysfunction of adolescent MDD as well as adult MDD.

In this study, adolescents with MDD showed decreased FA values in the left UF compared to HC. The UF connects the amygdala with inferior frontal regions including VPFC and ACC, which are key components of the frontal-limbic neural circuits involved in emotional processing (26, 28). Dysfunction of the amygdala-VPFC circuit have proved to play an important role in the pathophysiology of adolescent MDD (8, 27, 29, 30). The prior study with adult MDD have demonstrated decreased FA values in the dorsal part of the UF (31). In the recent DTI studies with adolescent MDD, Cullen et al. reported reduced FA values in the right UF (14), while LeWinn et al. reported lower FA and higher RD in bilateral UF (32). As in the medication-naïve adolescent MDD sample of our study, the current findings of decreased FA values in the left UF provide preliminary evidence that abnormal white matter structural integrity in amygdala-VPFC circuit may be present early in the unmedicated adolescent MDD and play an important role in the pathophysiology of adolescent MDD.

The major strength of our study is that multiple MRI methods (rs-fMRI and DTI) were used to detect functional connectivity, structural connectivity and their relationship in the same adolescent MDD sample. Our present results of decreased functional connectivity within amygdala-VPFC circuits as well as reduced structural connectivity between amygdala and VPFC in adolescent MDD suggest the potential association between functional and structural connectivity. Our explanation is that deficits of white matter integrity in the UF might contribute to the decrease of functional connectivity between amygdala and VPFC. We speculate that early abnormalities of brain development may be present in some adolescent MDD patients early in life, even before illness episodes. The unmaturation of brain development in adolescent may cause the inadequate compensatory mechanism, hence lead to the decrease of both functional and structural connectivity in adolescent MDD. The association between functional and structural disconnectivity in the amygdala-VPFC circuit may reflect the early onset mechanism of adolescent MDD, which need to be further investigated in future longitudinal studies including adolescents with high-risk of depression.

The limitation of our findings is that as no significant correlation between strength of functional connectivity and white matter integrity detected, the direct relationship between functional and structural connectivity in the amygdala-VPFC circuit within adolescent is still unclear. Since a borderline significant correlation was detected in the MDD group, our speculation is that the relative small sample size may limit our ability to detect the statistical significance in current study. Future studies with large sample size is important to further understand the relationship between functional and structural connectivity and the neurodevelopmental mechanism of adolescent MDD.

## Conclusions

Our study present the primary evidence of both functional and structural connectivity abnormalities of amygdala-VPFC circuit in the sample of first-episode medication-naïve adolescent MDD. The abnormal white matter structural integrity in adolescent MDD may reflect the unmaturation of early brain development in adolescent, which may cause the functional dis connectivity in the same circuit. Both functional and structural abnormalities of amygdala-VPFC circuit may present in the early onset of adolescent MDD and play an important role in the neuropathophysiology of adolescent MDD.

## Data Availability Statement

The datasets used and/or analyzed during the current study are available from the corresponding author on reasonable request.

## Ethics Statement

The studies involving human participants were reviewed and approved by the Medical Science Research Ethics Committee of the First Affiliated Hospital of China Medical University (approval reference number [2012]25-1). Written informed consent to participate in this study was provided by the participants’ legal guardian/next of kin.

## Author Contributions

FW, LK, and JS designed the study. JS, YZ, XJ, ZT, and HL acquired the data. LK, JS, and HG analyzed the data. FW, LK, JS, and ZT wrote the article.

## Funding

This study was supported by the National Natural Science Foundation of China (U1808204 and 81101012 to FW, 81301166 to LK), the Liaoning Scientific Foundation (2015020532 to FW and 201602833 to LK).

## Conflict of Interest

The authors declare that the research was conducted in the absence of any commercial or financial relationships that could be construed as a potential conflict of interest.

## Abbreviations

ACC, anterior cingulate cortex; AC–PC, anterior–posterior commissure; BA, Brodmann areas; DTI, diffusion tensor imaging; FA, fractional anisotropy; fMRI, functional magnetic resonance imaging; HAMD-17, The 17-item Hamilton Depression Rating Scale; HC, healthy control subjects; KSADS-PL, the Schedule for Affective Disorders and Schizophrenia for School-Age Children; MDD, major depressive disorder; MNI, Montreal Neurological Institute; PFC,prefrontal cortex; rs-fMRI, resting-state fMRI; rsFC, resting state functional connectivity; ROI, regions of interest; VPFC, ventral prefrontal cortex.
